# Biological and Immune Responses to Current Anti-SARS-CoV-2 mRNA Vaccines beyond Anti-Spike Antibody Production

**DOI:** 10.1155/2022/4028577

**Published:** 2022-05-14

**Authors:** Maurizio Federico

**Affiliations:** National Center for Global Health, Istituto Superiore di Sanità, Viale Regina Elena, 299, 00161 Rome, Italy

## Abstract

Several vaccine strategies are now available to fight the current SARS-CoV-2 pandemic. Those based on the administration of lipid-complexed messenger(m)RNA molecules represent the last frontiers in terms of technology innovation. mRNA molecules coding for the SARS-CoV-2 Spike protein are intramuscularly injected, thereby entering cells by virtue of their encapsulation into synthetic lipid nanovesicles. mRNA-targeted cells express the Spike protein on their plasma membrane in a way that it can be sensed by the immune system, which reacts generating anti-Spike antibodies. Although this class of vaccines appears as the most effective against SARS-CoV-2 infection and disease, their safety and efficiency are challenged by several factors included, but not limited to the following: emergence of viral variants, lack of adequate pharmacokinetics/pharmacodynamics studies, inability to protect oral mucosa from infection, and antibody waning. Emergence of viral variants can be a consequence of mass vaccination carried out in a pandemic time using suboptimal vaccines against an RNA virus. On the other hand, understanding the remainder flaws could be of some help in designing next generation anti-SARS-CoV-2 vaccines. In this commentary, issues regarding the fate of injected mRNA, the tissue distribution of the induced antiviral antibodies, and the generation of memory B cells are discussed. Careful evaluation of both experimental and clinical observations on these key aspects should be taken into account before planning booster administration, vaccination to non-at-risk population, and social restrictions.

## 1. Introduction

For many years, the use of RNA for therapeutics and vaccines was disregarded due to the supposed difficulties to be manipulated. The achievement of several technology improvements contributed to put this technique in the spotlight for its pharmaceutical use. These advancements included the use of base analogues, addition of a cap at the 5′ end, optimization of codon usage, and inclusion of untranslated elements at both 5′ and 3′ ends facilitating ribosome recognizing [1].

Starting from November 2011, the Biological Technology Office of the US Defense Department's Defense Advanced Research Project Agency invested large funding in several RNA-based vaccine programs. CureVac and Moderna companies were the first recipients of such financial supports. In the following years, biotech and Synthetic Genomics companies were funded by both public and private agencies, including NIH, Gates Foundation, Johnson & Johnson, Bayer, and Genentech to develop similar programs [2]. The disclosed investment of about 1 billion of dollars provided within a few years was the best witness of the enormous interest on the application of RNA vaccine technology to fight both tumors and infectious diseases.

Both preclinical and clinical data have proven both the feasibility and potentiality of the mRNA-based vaccine platform. Melanoma, triple-negative breast cancer, prostate cancer, and lung cancer were among the tumor diseases challenged by experimental mRNA vaccines in clinical trials. On the other hand, the same technique was evaluated against several viral diseases [2]. The anticipated success of this method in delivering any mRNA sequence into the cells represented the starting point towards the design of current mRNA-based anti-SARS-CoV-2 vaccines.

These vaccines are composed by “in vitro” synthesized mRNA molecules coding for full-length SARS-CoV-2 Spike glycoproteins from the ancestral strain (i.e., Wuhan isolate) in a prefusion conformation. The stabilization of the prefusion conformation is ensured by two consecutive proline substitutions at amino acid positions 986 and 987, at the top of the central helix of the S2 subunit. The mRNA molecules are complexed with lipids in a way that they are allowed to enter cells efficiently. In such a formulation, the mRNA molecules are expected to pass the plasma membrane of any kind of cell, thereby becoming available for translation by the cell cytoplasmic machinery. In humans, injections are usually carried out intramuscularly (i.m.) in the deltoid. Neosynthesized, full-length Spike proteins are anticipated to remain anchored to the host cell membrane in trimeric complexes, allowing the immune system to initiate the mechanisms leading to the development of anti-Spike adaptive immunity. It is supposed that local inflammation generated by coinjected substances and/or cellular responses to Spike expression may account for costimulation needed for an effective immune response.

Here, issues regarding pharmacokinetics/pharmacodynamics of mRNA vaccines, induced mucosal immunity, and antibody waning are analyzed. Careful evaluation of the weaknesses of current mRNA vaccines would be helpful for the generation of improved anti-SARS-CoV-2 immunogens.

## 2. Still Unresolved Aspects on Pharmacokinetics and Pharmacodynamics of mRNA-Based Vaccines

Several aspects regarding how the organism affects the fate of mRNA vaccines (pharmacokinetics), as well as how they influence the host physiology (pharmacodynamics), deserve thorough evaluation.

Concerning the fate of mRNA vaccines upon i.m. injection, their formulation implies that virtually any kind of cell can internalize the lipid-mRNA complexes. Clearly, i.m. inoculation favors the delivery of mRNA molecules into muscle cells. However, in view of the strong bioactivity of both mRNA and its translation products, monitoring possible additional sites of vaccine accumulation upon injection is of major relevance.

On this subject, results from a very accurate study carried out in cynomolgus macaques were published a few months before the pandemic outbreak [3]. It was clearly demonstrated that upon vaccine injection, both muscle cells and diverse types of immune cells express the protein coded by the mRNA. The authors reported that 4 hours after injection, mRNA molecules are internalized by immune cells at both the site of injection and proximal lymph nodes, in amounts appearing inversely proportional to the distance from the point of injection. After 28 hours, the levels of vaccine mRNA increased in lymph nodes, while decreasing in the injection site. Monocytes were found to be the immune cells most efficiently internalizing vaccine mRNA in both muscle tissues and lymph nodes. The latter ones were also found abundantly infiltrated by both B lymphocytes and dendritic cells expressing the mRNA vaccine.

A relevant message from this study is that mRNA does not localize at the inoculation site only, however spreading to proximal lymph nodes as early as 28 hours postinjection. Considering the observed rapidity of diffusion, the authors concluded that the spreading can be consequence of vaccine diffusion rather than cell migration, even if it is also conceivable that mRNA internalizing immune cells may reach lymph nodes through lymphatic circulation. When translated to current anti-SARS-CoV-2 mRNA vaccines, a direct consequence of mRNA internalization in immune cells can be that large amounts of the Spike protein would be persistently expressed in several districts of the body. The fate of SARS-CoV-2 Spike protein expressed by immune cells is essentially unknown. Hence, it is quite difficult to establish whether free circulation of Spike-expressing immune cells can be beneficial for the antiviral adaptive immune response. Conversely, membrane-associated Spike proteins can interact with cells expressing the ACE-2 receptor. This binding can perturb the functions of endothelial cells by inhibiting vital mitochondrial functions, leading to downstream endothelium pathology [4].

Vaccine mRNA molecules comprise 1-methyl-pseudouridine in place of uridine. The rationale for this change relied on the observation that after intravenous (i.v.) injection, decreased innate immune sensing together with increased mRNA stability and translation efficiency compared to unmodified mRNA has been observed [5]. Not consistently, more recent work emphasized that 1-methyl-pseudouridine modification of mRNA had no significant effect on both protein expression “in vivo” and mRNA immunogenicity compared to unmodified mRNA when it was delivered systemically. In particular, it was observed transient extracellular innate immune responses to modified mRNA included neutrophilia, myeloid cell activation, and upregulation of four serum cytokines, namely, CCL2, CCL5, CXCR9, and G-CSF [6].

After cell internalization, vaccine mRNA is expected to be translated by the cellular machinery until its intracellular degradation. Intriguingly enough, however, data from a recent study highlighted that subgenomic parts of SARS-CoV-2 RNA can integrate into DNA of human cells and patient-derived tissues [7]. Integrated SARS-CoV-2-related DNA sequences mostly originated from the 3′ end of the viral genome, which encompasses Spike-related sequences. Integrations of retrotranscribed SARS-CoV-2 sequences have been detected mostly in exon regions and correlated with the activity of the ubiquitous LINE-1 retrotransposon. Even if these findings have been questioned [8], implementing detailed genotoxicity studies in vaccinated subjects should be strongly recommended.

Once translated, vaccine-derived SARS-CoV-2 Spike is supposed to be embedded into the host cell membrane. However, based on the observations made with many other virus species, it is more than likely that at least part of the neosynthesized protein sheds and circulates into the body. Free circulating Spike protein might bind ACE-2-expressing cells, thereby inducing cell activation and damage [4], whose overall consequences depend on the levels of ACE-2 expression on the targeted tissues. When it occurs in blood vessels or heart tissues, consequent cell damage and inflammation can lead to vasculitis, pericarditis, and myocarditis [9–11].

Intracellular expression of vaccine mRNA leads to both vigorous and reproducible antibody response. In this context, possible effects induced by anti-idiotype antibodies should be taken in consideration, especially in recovered patients undergoing vaccination. Here, the amino acid sequences (referred to as idiotopes) in antigen-binding domains of anti-Spike antibodies can be immunogenic as consequence of the very high antibody levels induced. In this way, antibodies against these sequences (anti-idiotype antibodies) can be induced, and, as consequence of a molecular mimicry, part of them can bind the ligand of Spike protein, i.e., ACE-2 [12]. Thereby, physiologic functions of ACE-2 could be disturbed, for instance by blocking natural ligands or abnormally stimulating the receptor. Also, ACE-2-expressing cells binding anti-idiotype antibodies may undergo cell lysis through mechanisms mediated by the action of complement and/or immune cells. Notably, all these undesirable effects have the potential to occur after the disappearance of the Spike antigen. However, no evidences on the generation of anti-idiotype antibodies in SARS-CoV-2 vaccines have been produced so far.

In conclusion, several issues regarding both pharmacokinetics and pharmacodynamics of vaccine mRNA remain open. Deep investigations on them are mandatory to anticipate and, in this case, find countermeasures against both mid- and long-term side effects.

## 3. Anti-Spike Antibodies in Oral Mucosa

Subjects injected with current anti-SARS-CoV-2 vaccines develop anti-Spike antibodies with different specificities, including antireceptor binding domain (RBD), anti-N-terminal domain (NTD), and anti-S2 antibodies. Most part of neutralizing antibodies binds the RBD.

Among all immunoglobulin (Ig) classes, secretory IgAs are the most effective ones in protecting epithelial cells of mucosal surfaces from the attack of respiratory viruses [13]. In SARS-CoV-2 infected patients, the virus-neutralizing potency of IgAs was found superior compared to that of virus-specific IgGs detectable in both serum and saliva [14]. It was calculated that, upon infection, the levels of anti-RBD IgGs are about five times higher than those of anti-RBD IgA, however, being seven times less efficient in virus neutralization assays. Moreover, dimeric anti-RBD IgAs from oral/lung mucosa were found more potent than the monomeric counterpart detectable in serum. Unfortunately enough, the levels of RBD-specific IgA decay much more rapidly than IgGs [14].

Immunity to the oral mucosa is mandatory for any vaccine conceived to impede the diffusion of virus spreading through the oral routes. In consequence, evaluating the levels of virus-neutralizing IgA induced in the oral mucosa by anti-SARS-CoV-2 vaccines attracted the interest of many scientists with the intent to predict the vaccine effectiveness in limiting viral spread within humans.

Data published by Planas and colleagues demonstrated absence of neutralization activity in nasal swabs until 2 weeks after the second injection of mRNA vaccine despite good levels of binding activity (presumably due to the coexistence of anti-Spike IgGs) and in the presence of high titers of both binding and neutralizing antibodies in sera [15]. More recently, another paper reported the lack of anti-Spike IgA until three weeks after the second jab in the saliva of 43 health care workers producing very high levels of anti-Spike serum IgAs upon vaccination [16]. Furthermore, Roltgen and colleagues found minimal amounts of either anti-Spike-RBD and –N-terminal domain IgA antibodies in both serum and oral mucosa of vaccinated subjects seven weeks after the second injection [17]. In another couple of studies, a modest neutralizing capacity has been observed in saliva of mRNA vaccines [18, 19].

Taken together, these data indicate that differently from natural infection [20, 21], mRNA-dependent anti-SARS-CoV-2 vaccination seems unable to induce levels of oral immunity adequate to protect vaccines from replicating and transmitting infecting viruses. The obvious consequence, as also supported by real-world evidences, is that also vaccinees can be infected by SARS-CoV-2. Furthermore, the very recent demonstrations that the levels of viral replication in the mucosa of vaccinated and unvaccinated subjects are similar [22, 23] support the idea that vaccine-induced immunity is not able to block virus transmission.

## 4. Anti-Spike Antibody Waning and B Memory Cells

Antibody waning is a distinctive feature of the immune response against infection with SARS-CoV-2 as well as many other respiratory viruses. Unfortunately, it was reported that in COVID-19 patients, neutralizing antibodies have the most rapid decay kinetics among the different functional families of anti-Spike antibodies [24]. Accordingly, results from several groups are consistent with the idea that also vaccine-induced antibodies have a limited half-life. However, antibody waning would not be a major issue in case the vaccine would be able to generate a well-established B memory activity prompt to react as soon as the infecting virus is encountered. Despite a limited number of studies suggesting that mRNA vaccines can generate anti-Spike memory B cells (MBCs), however, their absolute number and, most importantly, tissue distribution pose relevant questions.

Studies on SARS-CoV-2-specific MBCs induced by vaccines are sparse and refer uniquely to cells isolated from peripheral blood. Goel and colleagues measured levels of RBD-specific MBCs (i.e., B cells having the potential to secrete neutralizing antibodies) 1 week after the second injection. By flow cytometric analysis, they found a mean of 0.1% of specific cells over the total of MBCs, which comprised 0.3% of Spike-specific MBCs for the most part expressing IgG [25]. Another study compared the number of MBCs specific for RBDs from the ancestral virus isolate with that specific for RBDs of a number of variants of concern (VoCs) two weeks after the second vaccine injection [26]. After three-day “in vitro” stimulation of PBMCs with human recombinant IL-2 and R848, a maximum of 0.015% specific over the total of cells was detected by B cell EliSpot analysis, with reduced percentages in the case of RBDs from VOCs. Mortari and colleagues found a peak of RBD-positive MBCs 7 days after the second injection, slightly decreasing 62 days thereafter. At this time, flow cytometry analysis estimated the percentages of RBD-specific MBCs around 0.01% of total CD19^+^ CD24^+^ CD27^+^ CD38^−^ cells, in the presence of 15% of MBCs showing high affinity to trimeric Spike [16]. Sokal and colleagues reported barely detectable RBD-specific MBCs within PBMCs of double vaccinated subjects 2 months after boosting [27].

Taken together, these data seem not sufficient to establish whether current mRNA vaccines can generate an adequate, durable, and reactivatable humoral response after antibody waning. Most importantly, in view of the strict compartmentalization of the lung immune system, additional studies are needed to evaluate the immune memory at the level of both high and low respiratory compartments.

In fact, the development of lung immune memory is largely not influenced by events occurring in both the peripheral circulation and lymphoid organs. In many instances, lymphocytes in the lungs are maintained independently of the pool of circulating lymphocytes, and their continuous loss through intraepithelial migration towards the airways is constantly replenished by homeostatic proliferation. Recently, a seminal study on a murine model susceptible to influenza virus infection demonstrated the presence of lung-resident memory B cells (BRMCs) as the major memory effectors of humoral antiviral immunity [28]. BRMCs are phenotypically distinguishable from MBCs from lymphoid tissues by virtue of high CXCR3 levels and absence of CD62L. Formation of BRMCs requires encounter with antigens in the lung, where they can differentiate. Antigen-specific BRMCs are maintained in the lung and do not recirculate and respond to infection/reinfection very rapidly. All these key observations have been very recently confirmed in mice infected with *Streptococcus pneumoniae* [29]. Of major interest, data from the same paper demonstrated the presence of B memory cells showing the BRMC phenotype in human lungs. Consistently, Weisel and colleagues identified tissue-resident BRMCs in the human gut [30].

Blocking virus replication in the oral mucosa represents a key step in the fight against SARS-CoV-2 spread. Optimal levels of both humoral and cellular immunity should be achieved locally at the viral port of entry, rather than peripherally. Based on the here above summarized observations, it is more than desirable that new antibody-based anti-SARS-CoV-2 vaccines designed to induce effective and long-lived protection would be delivered to the respiratory tract. This argument is strengthened also by the evidence that mucosal anti-SARS-CoV-2 dimeric IgAs are several fold more potent that the serum-derived monomeric counterpart [14, 31]. In addition, oral delivery of new immunogens/vaccines has the potential to generate very limited systemic side effects even after repeated administrations.

Taken together, these observations strongly support the idea that orally administrated vaccines would represent a relevant amelioration compared to current ones, which generate an immunity not strong enough to impede virus replication in the oral mucosa and transmission [32].

## 5. Possible Consequences of Repeated Vaccinations Using the Same Immunogen

Systemic administration of vaccines against respiratory viruses often associates with unsatisfactory outcomes. For instance, the efficacy of seasonal anti-influenza vaccines rarely overcomes 50% of protection.

The immunological correlates of protection against SARS-CoV-2 infection are still unknown, where the term “correlate of protection” refers to a laboratory parameter associated with protection from a clinical disease [33]. A coordinated action of CD4^+^ T cells, CD8^+^ T cells, and neutralizing antibodies seems necessary to control SARS-CoV-2 infection. In this scenario, neutralizing antibodies certainly play a key role in protecting from infection. However, in the case of current vaccines, which exclusively rely on humoral immunity, antibody waning and uncertain BMC effectiveness in the lung tissue represent not easily surmountable limitations. It is not obvious to predict whether additional vaccine inoculations could improve the quality, intratissue distribution, and relative duration of the immune response. It has been observed that repeated antigen encountering can select for plasmablasts/plasmacells producing antibodies with increasing affinity, as also demonstrated for SARS-CoV-2 infections [34–36]. However, one should consider that this phenomenon could occur also for nonneutralizing antibodies, some of which might have pathogenic effects.

**Figure 1:**
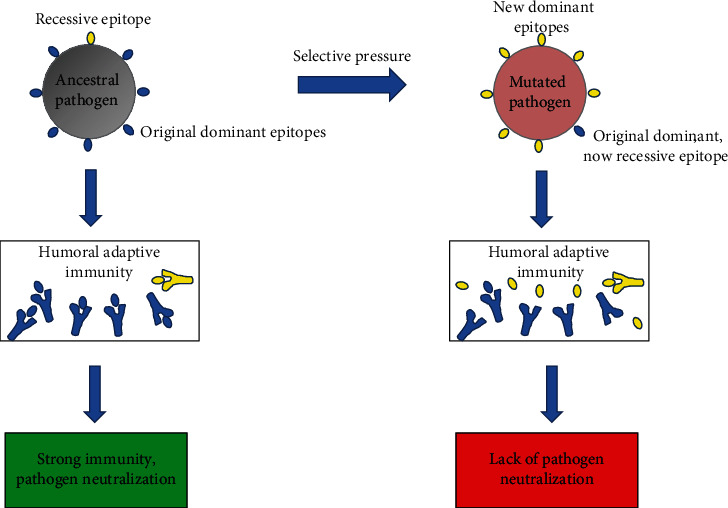
The “original antigenic sin.” When the body first encounters a pathogen, it produces effective antibodies against its dominant antigen and thus eliminates the pathogen. Selective pressure can originate pathogens with new dominant antigens, with the original antigens now being recessive. In this case, the immune system still produces the former antibodies against the old “now recessive antigen” and develops antibodies against the new dominant one scarcely. The results are the production of ineffective antibodies and generation of weak immunity.

Virus replication in the context of suboptimal antiviral action of vaccine-induced antibodies can lead to emergence of resistant virus quasispecies. In the SARS-CoV-2 case, this process could have contributed to the selection of VoCs, whose rapid emergence paralleled mass vaccination. This phenomenon can affect the anticipated outcomes from additional vaccine cycles. In fact, it is well known that repeated vaccinations against pathogen evolving mutants like SARS-CoV-2 have the risk to meet with the phenomenon referred to as “original antigenic sin” [37]. In detail, the humoral immune response elicited against immunodominant epitopes of the first pathogen generates a sort of “immune imprinting” in a way that the response against subsequent infection with a mutated form of the pathogen cannot be able to recognize new emerging immunodominant epitopes. As a result, either reduced or no protection against the new pathogenic strain can be generated (Figure 1).

Intriguingly enough, very recently, it has been reported that a third-dose booster with an Omicron-based mRNA vaccine on macaques previously treated with two doses of mRNA-1273 vaccine (based on the ancestral Spike sequence) did not offer advantages in terms of protection against infection with the Omicron VoC respect to the homologous booster [38]. Consistent with the “antigenic original sin” theory, it is conceivable that the immune system previously educated by the two mRNA-1273 vaccine doses reacted to the Omicron-based third dose still producing Abs mainly directed to the ancestral S protein.

## 6. Conclusions

Current mRNA-based anti-SARS-CoV-2 vaccines have provided protection. However, antibody waning, unsatisfactory mucosal immunity, and the “antigenic original sin” mechanisms should be adequately evaluated at the time of decision to proceed towards additional cycles of vaccination. In fact, challenging an emerging VoC (as in the case of the widespread Omicron variants) with repeated injections of vaccines designed for a virtually disappeared virus quasispecies may be not the best strategy, as also suggested by the strong decrease of vaccine effectiveness calculated after the fourth dose (Table 1) [39, 40]. On the other hand, the likelihood of side effect occurrence increases with the number of injections.

For all these reasons, repeated mass vaccination of non-at-risk populations, including infants and adolescents, asks for much more extended and careful evaluation of both mid- and long-term risks.

## Figures and Tables

**Table 1:** Effectiveness against the SARS-CoV-2 Omicron (1.1.529) variant after 2 to 4 vaccine doses^a^.

Ref	Vaccine type	no. of doses	Time after last dose (weeks)				
			2-4	5-9	10-14	15-19	≥25
[39]	BNT162b2	2	65.5%	48.7%	30.1%	15.4%	8.8%
[39]	BNT162b2	3	67.2%	55%	45.7%		
[40]	BNT162b2	4	30%				
[39]	mRNA-1273	2	75.1%	52.8%	35.6%	25.3%	14.9%
[39]	mRNA-1273	3	66.3%	64.4%			

^a^Cumulative data as calculated on populations from either England or Israel (for the 4^th^ dose only).

## Abbreviations

ACE-2: Angiotensin-converting enzyme 2
BRMCs: Resident B memory cells
COVID-19: Coronavirus disease 2019
Ig: Immunoglobulin
LINE: Long interspersed nuclear element
MBCs: Memory B cells
mRNA: Messenger RNA
RBD: Receptor-binding domain
SARS-CoV-2: Severe acute respiratory syndrome coronavirus 2
VoC: Variant of concern.

## References

[1] Sahin U., Karikó K., Türeci Ö. (2014). MRNA-based therapeutics-developing a new class of drugs. *Nature Reviews. Drug Discovery*.

[2] DeFrancesco L. (2017). The 'anti-hype' vaccine. *Nature Biotechnology*.

[3] Lindsay K. E., Bhosle S. M., Zurla C. (2019). Visualization of early events in MRNA vaccine delivery in non-human primates via PET-CT and near-infrared imaging. *Nature Biomedical Engineering*.

[4] Lei Y., Zhang J., Schiavon C. R. (2021). SARS-CoV-2 spike protein impairs endothelial function via downregulation of ACE 2. *Circulation Research*.

[5] Karikó K., Muramatsu H., Welsh F. A. (2008). Incorporation of pseudouridine into MRNA yields superior nonimmunogenic vector with increased translational capacity and biological stability. *Molecular Therapy*.

[6] Kauffman K. J., Mir F. F., Jhunjhunwala S. (2016). Efficacy and immunogenicity of unmodified and pseudouridine-modified MRNA delivered systemically with lipid nanoparticles in vivo. *Biomaterials*.

[7] Zhang L., Richards A., Barrasa M. I., Hughes S. H., Young R. A., Jaenisch R. (2021). Reverse-transcribed SARS-CoV-2 RNA can integrate into the genome of cultured human cells and can be expressed in patient-derived tissues. *Proceedings of the National Academy of Sciences of the United States of America*.

[8] Parry R., Gifford R. J., Lytras S., Ray S. C., Coin L. J. M. (2021). No evidence of SARS-CoV-2 reverse transcription and integration as the origin of chimeric transcripts in patient tissues. *Proceedings of the National Academy of Sciences of the United States of America*.

[9] Mücke V. T., Knop V., Mücke M. M., Ochsendorf F., Zeuzem S. (2021). First description of immune complex vasculitis after COVID-19 vaccination with BNT162b2: a case report. *BMC InfectiousDiseases*.

[10] Fatima M., Ahmad Cheema H., Ahmed Khan M. H. (2022). Development of myocarditis and pericarditis after COVID-19 vaccination in adult population: a systematic review. *Annals of Medicine and Surgery*.

[11] Oster M. E., Shay D. K., Su J. R. (2022). Myocarditis cases reported after mRNA-based COVID-19 vaccination in the US from December 2020 to August 2021. *JAMA*.

[12] Murphy W. J., Longo D. L. (2022). A possible role for anti-idiotype antibodies in SARS-CoV-2 infection and vaccination. *The New England Journal of Medicine*.

[13] Mazanec M. B., Coudret C. L., Fletcher D. R. (1995). Intracellular neutralization of influenza virus by immunoglobulin A anti-hemagglutinin monoclonal antibodies. *Journal of Virology*.

[14] Sterlin D., Mathian A., Miyara M. (2021). IgA dominates the early neutralizing antibody response to SARS-CoV-2. *Science Translational Medicine*.

[15] Planas D., Bruel T., Grzelak L. (2021). Sensitivity of infectious SARS-CoV-2 B.1.1.7 and B.1.351 variants to neutralizing antibodies. *Nature Medicine*.

[16] Mortari E. P., Russo C., Vinci M. R. (2021). Highly-specific memory B cells generation after the 2 ^nd^ dose of BNT162b2 vaccine compensate for the decline of serumantibodies and absence of mucosalIgA; preprint. *Cells*.

[17] Roltgen K., Nielsen S. C. A., Arunachalam P. S. (2021). MRNA vaccination compared to infection elicits an IgG-predominant response with greater SARS-CoV-2 specificity and similar decrease in variant Spike recognition. *medRxiv*.

[18] Sheikh-Mohamed S., Chao G. Y. C., Isho B. (2021). A mucosal antibody response is induced by intra-muscular SARS-CoV-2 MRNA vaccination. *medRxiv*.

[19] Ketas T. J., Chaturbhuj D., Portillo V. M. C. (2021). Antibody responses to SARS-CoV-2 MRNA vaccines are detectable in saliva. *Pathogens and Immunity*.

[20] Ravichandran S., Grubbs G., Tang J. (2021). Systemic and mucosal immune profiling in asymptomatic and symptomatic SARS-CoV-2–infected individuals reveal unlinked immune signatures. *Science Advances*.

[21] Varadhachary A., Chatterjee D., Garza J. (2020). Salivary anti-SARS-CoV-2 IgA as an accessible biomarker of mucosal immunity against COVID-19. *medRxiv*.

[22] Mostafa H. H., Luo C. H., Morris C. P. (2021). SARS-CoV-2 infections in MRNA vaccinated individuals are biased for viruses encoding Spike E484K and associated with reduced nfectious virus loads that correlate with respiratory antiviral IgG Levels. *medRxiv*.

[23] Acharya C. B., Schrom J., Mitchell A. M. (2021). No significant difference in viral load between vaccinated and unvaccinated, asymptomatic and symptomatic groups infected with SARS-CoV-2 delta variant. *medRxiv*.

[24] Dispinseri S., Secchi M., Pirillo M. F. (2021). Neutralizingntibodyresponses to SARS-CoV-2 in symptomatic COVID-19 ispersistent and critical for survival. *Nature Communications*.

[25] Goel R. R., Apostolidis S. A., Painter M. M. (2021). Distinct antibody and memory B cell responses in SARS-CoV-2 naïve and recovered individuals after MRNA vaccination. *Science Immunology*.

[26] Haralambieva I. H., Monroe J. M., Ovsyannikova I. G., Grill D. E., Poland G. A., Kennedy R. B. (2021). Homologous and variant-specific memory B-cell and antibody responses after SARS-CoV-2 MRNA vaccination. *medRxiv*.

[27] Sokal A., Chappert P., Barba-Spaeth G. (2021). Maturation and persistence of the anti-SARS-CoV-2 memory B cell response. *Cell*.

[28] Allie S. R., Bradley J. E., Mudunuru U. (2019). The establishment of resident memory B cells in the lung requires local antigen encounter. *Nature Immunology*.

[29] Barker K. A., Etesami N. S., Shenoy A. T. (2021). Lung-resident memory B cells protect against bacterial pneumonia. *The Journal of Clinical Investigation*.

[30] Weisel N. M., Weisel F. J., Farber D. L. (2020). Comprehensive analyses of B-cell compartments across the human body reveal novel subsets and a gut-resident memory phenotype. *Blood*.

[31] Wang Z., Lorenzi J. C. C., Muecksch F. (2021). Enhanced SARS-CoV-2 neutralization by dimeric IgA. *Science Translational Medicine*.

[32] Bleier B. S., Ramanathan M., Lane A. P. (2021). COVID-19 vaccines may not prevent nasal SARS-CoV-2 infection and asymptomatic transmission. *Otolaryngology and Head and Neck Surgery*.

[33] Plotkin S. A. (2008). Vaccines: correlates of vaccine-induced immunity. *Clinical Infectious Diseases*.

[34] Sokal A., Barba-Spaeth G., Fernández I. (2021). Memory B cells control SARS-CoV-2 variants upon MRNA vaccination of naive and COVID-19 recovered individuals. *bioRxiv*.

[35] Gaebler C., Wang Z., Lorenzi J. C. C. (2021). Evolution of antibody immunity to SARS-CoV-2. *Nature*.

[36] Rodda L. B., Netland J., Shehata L. (2021). Functional SARS-CoV-2-specific immune memory persists after mild COVID-19. *Cell*.

[37] Zhang A., Stacey H. D., Mullarkey C. E., Miller M. S. (2019). Original antigenic sin: how first exposure shapes lifelong anti-influenza virus immune responses. *Journal of Immunology*.

[38] Gagne M., Moliva J. I., Foulds K. E. (2022). MRNA-1273 or MRNA-Omicron boost in vaccinated macaques elicits comparable B cell expansion, neutralizing antibodies and protection against Omicron. *BioRxiv*.

[39] Andrews N., Stowe J., Kirsebom F. (2022). Covid-19 vaccine effectiveness against the Omicron (B.1.1.529) variant. *The New England Journal of Medicine*.

[40] Regev-Yochay G., Gonen T., Gilboa M. (2022). Efficacy of a fourth dose of Covid-19 MRNA vaccine against Omicron. *The New England Journal of Medicine*.

[41] Federico M. https://www.researchgate.net/publication/356909387_Biological_and_immune_responses_to_current_anti-SARS-_CoV-2_mRNA_vaccines_beyond_anti-Spike_antibody_production/.

